# Phase I metabolic profiling and unexpected reactive metabolites in human liver microsome incubations of X-376 using LC-MS/MS: bioactivation pathway elucidation and *in silico* toxicity studies of its metabolites

**DOI:** 10.1039/c9ra09115g

**Published:** 2020-02-03

**Authors:** Mohamed W. Attwa, Adnan A. Kadi, Ali S. Abdelhameed

**Affiliations:** Department of Pharmaceutical Chemistry, College of Pharmacy, King Saud University P.O. box 2457 Riyadh 11451 Kingdom of Saudi Arabia asaber@ksu.edu.sa +966 1146 76 220 +966 1146 70237; Students' University Hospital, Mansoura University Mansoura 35516 Egypt

## Abstract

X-376 is a novel new generation anaplastic lymphoma kinase (ALK) inhibitor that can cross the blood brain barrier, so it can be used in patients with both ALK-positive NSCLC with CNS metastases. In this study, X-376 was incubated with human liver microsomes, and *in vitro* phase-I metabolic reactions were performed to generate X-376 reactive intermediates, which cannot be detected directly as they are unstable. Utilizing LC-MS/MS, we characterized X-376 metabolites and checked for the reactive electrophile generation using nucleophilic trapping agents, namely, potassium cyanide and GSH, which form stable adducts for characterization by mass spectrometry. Four X-376 phase-I metabolites and three reactive intermediates (one quinone methide and two iminium ions) were characterized and the bioactivation pathways were proposed. X-376 bioactivation occurred through unexpected novel pathways. Although the pyridazine ring was found to be bioactivated, no bioactivation was detected in the expected *N*-methyl piperazine ring that is often found for similar structures. The dichloro-phenyl group was bioactivated by a novel mechanism that was verified by LC-MS/MS. We propose that the X-376 reported side effects may be due to the formation of reactive metabolites. The *in silico* toxicity assessment of X-376 metabolites was carried out using DEREK software and structural modification were proposed to reduce their side effects and to validate the bioactivation pathway theory using StarDrop DEREK module. Further drug discovery studies can be done according to this concept, thus allowing the development of new drugs with more safety profile that was confirmed by using StarDrop software. To our knowledge, this is the first study of *in vitro* metabolic profiling or structural characterization and toxicological properties of the generated metabolites for X-376.

## Abstract 2

Metabolites of X-376 were characterized by LC-MS/MS. Pyridazine ring and dichloro-phenyl groups were bioactivated by novel pathways.

## Graphical abstract

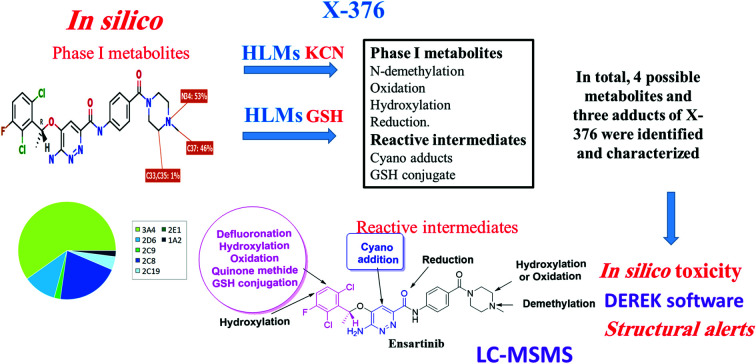

## 1. Introduction

Cancer is associated with high mortality, causing approximately one fourth of incident deaths worldwide.^1^ Lately, the treatment strategies based on molecular targeting have been utilized in cases of disseminated cancer, depending on the identified tumor suppressor oncogenes and genes that participate in the patient's cancer progression.^2^ Among all cancer types, lung cancer is considered to have the highest mortality rate worldwide. In 2012, lung cancer caused 1.59 million deaths, which represents 20% of all cancer deaths.^3^ Non-small cell lung cancers (NSCLCs) represent 90% of all lung cancers and involve numerous subtypes driven by several activated oncogenes.^4,5^ The treatment of NSCLC patients greatly improved when the targeted personalized therapies were used.^6–8^ Anaplastic lymphoma kinase (ALK) belongs to the insulin receptor tyrosine kinase family (RTK).^9^ ALK inhibitors are antineoplastic agents that act on tumors with rearrangements in ALK genes such as the EML4-ALK gene fusion.^10^ EML4-ALK translocations have been found in 4–7% of NSCLC cases.^11^

The first ALK inhibitors generation (*e.g.*, Crizotinib) has become less effective due to resistant mutations in NSCLCs that have become treatable by the development of a new ALK inhibitor generation (*e.g.*, brigatinib, X-376 and X-396 (ensartinib)).^12–14^ Compared with crizotinib, X-376 and X-396 inhibit ALK with greater potency in biochemical assays (10-folds) and cell-based assays (3- to 10-folds) with additional activity as MET inhibitors. Both X-376 and X-396 are being developed by Xcovery.^15^X-376 is considered to be a novel, more potent, and specific new generation ALK TKI with potential therapeutic relevance. It displays synergistic antitumor activity when combined with the mTOR inhibitor, rapamycin. It can also cross the blood brain barrier, so it can be used in patients with both ALK-positive NSCLC and CNS metastases.^12^

Detoxification of drugs through metabolic reactions that increase the hydrophilicity of endogenous compounds and xenobiotics occurs to aid excretion outside the human body. The formed metabolites often exhibit low toxicity in comparison to the precursor molecules. Though, in special cases, the generation of reactive intermediates occurs that exhibit more toxicity.^16–18^ Reactive intermediates are electron deficient, thus, they can modify proteins and DNA through covalent bonds, which is the initial step in drug-mediated organ toxicities.^19,20^ The detection of reactive metabolite formation is an important task in understanding drug-induced toxicity. Reactive intermediates are usually generated by phase-I metabolic reactions and cannot be identified directly as they are unstable in nature. So, a trapping nucleophile can be utilized to interact with the reactive intermediates and capture the newly formed adducts, which are stable and suitable for characterization by LC-MS/MS.^21,22^

**Fig. 1:**
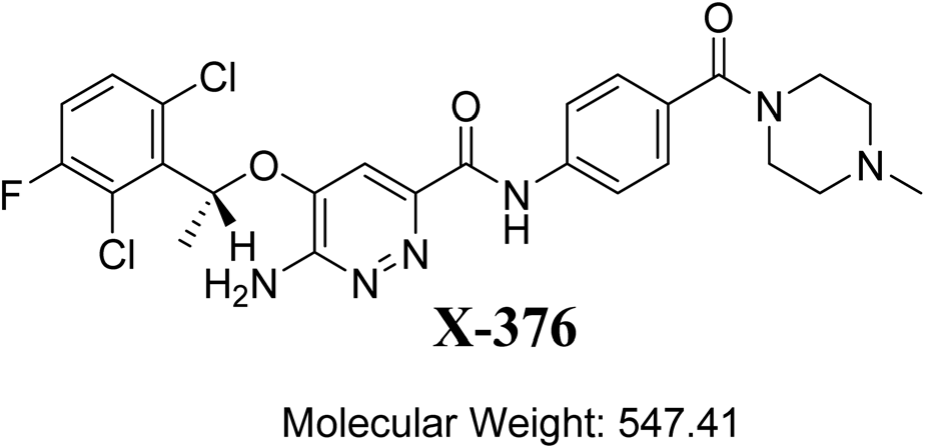
Chemical structure of X-376.

The designation of X-376 according to the International Union of Pure and Applied Chemistry is 6-amino-5-[(1∼{*R*})-1-(2,6-dichloro-3-fluorophenyl)ethoxy]-∼{*N*}-[4-[(3∼{*R*},5∼{*S*})-3,5-dimethylpiperazine-1-carbonyl]phenyl]pyridazine-3-carboxamide (Fig. 1). The chemical structure of X-376 involves cyclic tertiary amine rings (piperazine) and a dichloro-fluorophenyl ring. The piperazine ring can undergo bioactivation by iminium ion formation that can be captured using potassium cyanide.^23–26^ The dichloro-fluorophenyl ring can perform bioactivation by forming a quinone derivative that can be trapped by glutathione (GSH).^27^ The adducts formed through nucleophilic–electrophilic reactions are considered stable, and can be isolated and identified by LC-MS/MS.^21–23,28,29^ We hypothesize that these reactive electrophiles potentially participate in the reported side effects of X-376.

In this study, we utilized LC-MS/MS for *in vitro*X-376 metabolites' characterization that were formed on incubation with HLMs and then tested the generation of reactive electrophiles using trapping agents. Importantly, we found that X-376 bioactivation occurred through unexpected novel pathways, which was confirmed using *in vitro* laboratory work and *in silico* software. This could be a new strategy for reducing the side effects of newly developed drugs without affecting their pharmacological activity, which was proposed using StarDrop software. Knowing the bioactive center and structural alerts in the X-376 structure helped in making the targeted modifications to improve its safety and retain its efficacy, which were confirmed using DEREK software. Four *in vitro* phase I metabolites, two cyano adducts, and one GSH conjugate of X-376 were characterized. The proposed pathways involved oxidation, reduction, *N*-demethylation, and hydroxylation. The *in silico* toxicity assessment of X-376 metabolites was carried out using DEREK software and structural modifications were proposed to reduce their side effects and to validate the bioactivation pathway theory using StarDrop software.

## 2. Chemicals and methods

### 2.1. Chemicals

Chemicals and reagents, including their sources, are mentioned in Table 1.

**Table 1:** List of materials

Name^a^	Source (USA)
X-376	Med Chem. Express company
Pooled human liver microsomes (HLMs, M0567), acetonitrile, ammonium formate, potassium cyanide, GSH, and formic acid	Sigma-Aldrich company
HPLC-grade water (H_2_O)	Milli-Q Plus Purification System

a Reference powders and solvents are analytical grade (AR).

### 2.2. Chromatography conditions

An Agilent 6410 triple quadrupole fitted to an electrospray ionizer (ESI) with an Agilent 1200 rapid resolution liquid chromatographer (RRLC) was used. The optimized chromatographic and mass parameters were selected for X-376 and its related metabolites. Fragment ions for X-376 and its metabolites were formed inside the second hexapole (collision cell) by collision induced dissociation technique using high purity nitrogen. The selected chromatographic parameters for the separation of X-376 metabolites are shown in Table 2. The LC-MS/MS analytical parameters were adjusted to achieve the optimum resolution of X-376 metabolites with good sensitivity (Table 1). Slow gradient system with long run time was developed to separate X-376, phase I metabolites, and reactive metabolite adducts. A triple quadrupole (QqQ) mass analyzer, operated in the positive charge mode with an ESI, was utilized for estimation. Nitrogen gas (11 L min^−1^) was used for drying of spray in the ESI source and for collision (55 psi) for fragmentation studies in the collision cell. Flow injection analysis was utilized to optimize all the mass spectrometric parameters to achieve the highest ion intensity. The values of ESI temperature (*T*) and capillary voltage (*V*) were set at 350 °C and 4000 V, respectively. Data acquisition was managed using the Mass Hunter software. The identification of X-376 metabolites was done using product ion (PI) scan mode for the mass transitions (parent to daughter ions). The fragmentor voltage (FV) was adjusted at 145 V with collision energy (CE) of 30 eV for X-376 metabolites. PI mode was used for structural identification to discard any interference from the HLM matrix constituents and to elevate the LC-MS/MS method sensitivity.

**Table 2:** LC-MS/MS chromatographic parameters

LC			MS/MS	
LC	Agilent RRLC 1200		MS	Agilent 6410 Triple Quadrupole
Gradient mobile phase	A: 5 mM ammonium formate in H_2_O		ESI	Positive ESI
	B: acetonitrile			Drying nitrogen gas
	0.3 mL min^−1^			11 L min^−1^ flow rate
	65 min			55 psi
C_18_ column (Agilent Eclipse Plus)	150 mm length			350 °C
	2.1 mm ID			4000 V capillary voltage
	3.5 μm particle size		Modes	MS scan and PI
	23 ± 2 °C		Collision gas	High-purity nitrogen gas
Gradient mobile phase	Time	% Acetonitrile	Drug	X-376 and its related metabolites
	0	5		
	5	5	Mass analyzer	30 eV collision energy
	40	60		200 ms dwell time
	60	90		500 ms scan time
	65	5		145 V fragmentor voltage

### 2.3. *In silico* prediction of X-376 metabolites using P450 metabolism module of StarDrop software

Our objective was to identify the vulnerability of key sites of metabolism, as revealed by decreasing site lability that was indicated by composite site lability (CSL) and also the predicted regioselectivity of metabolism by the major isoforms supposed to be responsible for metabolism. The results from the WhichP450™ model are shown by the pie chart used for indication of the most likely cyp450 isoform that has a major role in X-376 metabolism.

### 2.4. *In silico* prediction of the toxicity of X-376 metabolites and reactivity using DEREK software

The screening for the predicted toxicity of X-376 metabolites was performed using DEREK software, which was also utilized to screen the structural alerts for X-376 so as to propose structural modifications at the supposed bioactive centres that stop the toxicity sequence.

### 2.5. HLM incubation

The detection of X-376 generated metabolites was done by incubation of 30 μM X-376 with 1.0 mg mL^−1^ HLMs in 50 mM phosphate buffer (pH 7.4), which contains 3.3 mM MgCl_2_ in a total volume of 1 mL in a thermostatic shaking water bath (37 °C for 120 min). The X-376 metabolic pathway began by addition of 1.0 mM NADPH and terminated by the addition of 2 mL acetonitrile (ice-cold), which was also utilized as a precipitating agent of excess protein that was removed by 9000×*g* centrifugation (15 min, 4 °C). The supernatants were translocated to tubes for evaporation to dryness under a stream of nitrogen. Fifteen microliters of each reconstituted sample (residue in mobile phase) were analyzed using LC-MS/MS.^30,31^

### 2.6. Identification of X-376 reactive metabolites

After a full MS scan over a specific range, the extracted ion chromatograms of selected molecular ion peaks (MIP) were used to locate the expected metabolites in the total ion chromatograms (TIC). Peak fractions containing the product ions with predicted identity were subjected to fragmentation using the product ion mode to generate daughter ions (DIs), which were needed to reconstruct the chemical structures of the original molecular ion. Thus, the fragmentation pattern of X-376 was utilized as a guide for the interpretation and confirmation of the supposed chemical structures of the reactive and *in vitro* metabolites formed during X-376 metabolism. X-376 HLM incubation was repeated using similar conditions by adding GSH or potassium cyanide for trapping the bioactive intermediates. To verify the results, all the experiments were repeated three times using controls (without HLMs or NADPH).

## 3. Results and discussion

### 3.1. Results of the *in silico*X-376 metabolites' prediction

**Fig. 2:**
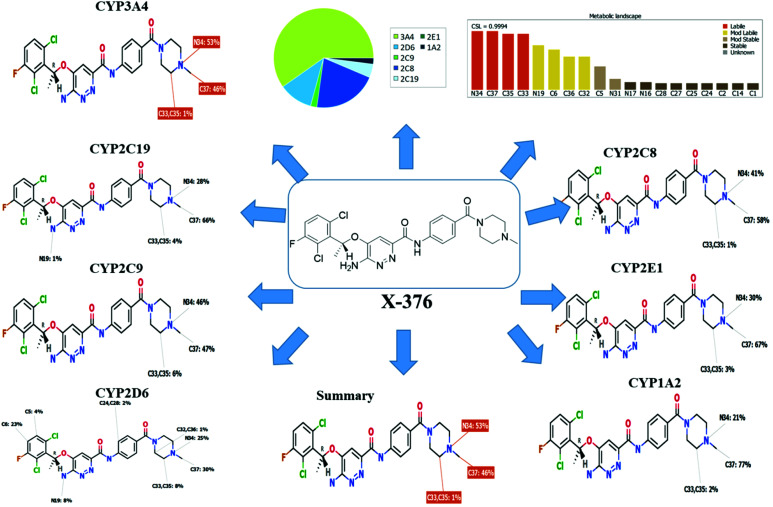
Proposed metabolic sites for X-376 by StarDrop WhichP450™ model.

The metabolic landscape for X-376 indicates the lability of each site with respect to metabolism by CYP3A4 in absolute terms so as to guide the prediction of X-376 metabolites and also the optimization of chemical structures for improving the metabolic stability. This indicates that N34, C37, C33, and C35 on the *N*-methyl piperazine are predicted to be the most labile sites of metabolism, which matched with the experimental results. The CSL is shown at the top-right of the metabolic landscape. The results from the WhichP450™ model are shown in the pie chart used for the indication of the most likely cyp450 isoform that has a major role in X-376 metabolism (Fig. 2). Cyp3A4 was found to have a major role in X-376 metabolism.

### 3.2. Results of *in silico*X-376 structural alert sites and toxicity prediction

**Fig. 3:**
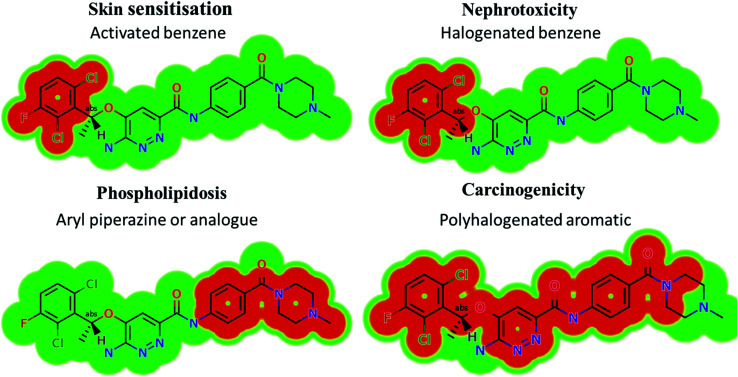
DEREK outcomes showing structural alerts with the proposed side effects of X-376. Red color indicates the structural alerts.

Based on the *in silico* predictions and the knowledge from literature, a list of probable metabolites and reactive intermediates was set. The expected potential atomic sites for the bioactivation and cyanide attack in the X-376 chemical structure are on two α carbon atoms adjacent to the nitrogen atoms of the piperazine ring (Fig. 3). The *in silico* toxicity assessment of X-376 metabolites was carried out using DEREK software; structural modifications were proposed to reduce their side effects and to validate the bioactivation pathway theory using StarDrop software (Fig. 3).

X-376 and its metabolites show structural alerts, as seen in Fig. 3, which gave rise to the proposed sites, as listed in Table 3. M3 and M4 did not exhibit phospholipidosis due to the presence of the oxidized piperazine ring. Groups that provide steric hinderance such as methyl (ensartinib) or oxygen (M3 and M4) stop the toxicity, as proposed by DEREK software. X-376 and its metabolites show nephrotoxicity, carcinogenicity, and skin sensitization due to halogenated benzene, polyhalogenated aromatic, and activated benzene moieties. Table 3 shows the complete list of *in vitro*X-376 metabolites with DEREK results for the proposed toxicity profile. Small structural modification at the α carbon to N34 stopped the phospholipidosis toxicity (Fig. 3).

**Table 3:** Qualitative toxicity prediction of X-376 and its metabolites by DEREK analysis

X-376 and its metabolites	Phospholipidosis	Nephrotoxicity	Carcinogenicity	Skin sensitization	Chromosome damage, teratogenicity, genotoxicity, and mutagenicity
	Aryl piperazine	Halogenated benzene	Polyhalogenated aromatic	Activated benzene	
X-376	Plausible	Equivocal	Plausible	Plausible	NA
M1	Plausible	Equivocal	Plausible	Plausible	NA
M2	Plausible	Equivocal	Plausible	Plausible	NA
M3	NA	Equivocal	Plausible	Plausible	NA
M4	NA	Equivocal	Plausible	Plausible	NA

### 3.3. Identification of X-376 related metabolites

Four X-376 metabolites were generated by four phase-I metabolic pathways (oxidation, *N*-demethylation, reduction, and hydroxylation). Two cyano adducts and one GSH adduct were identified (Table 4).

**Table 4:** *In vitro* phase-I and reactive metabolites of X-376

	MS scan	Most abundant fragment ions	Rt.^a^ (min)	Metabolic reaction
X-376	547	447, 357, 257, 138	38.9	Main drug

**Phase-I metabolites**				
M1	533	447, 343, 257, 138	38.0	*N*-Demethylation at the piperazine ring
M2	549	449, 359, 259, 139	35.6	Reduction metabolic reaction in the amide group attached to the pyridazine ring
M3	561	447, 371, 257, 138	44.1	Oxidation at the piperazine ring
M4	563	447, 373, 257, 138	42.0	Hydroxylation at the piperazine ring

**Reactive metabolites**				
M5	572	472, 282, 120, 104	39.2	Cyano addition at the pyridazine ring
M6	588	472, 282, 104	42.9	Hydroxylation at the piperazine ring and cyano addition at the pyridazine ring
M7	852	496, 357, 257, 176	28.9	Defluorination, hydroxylation, oxidation, and GSH conjugation

a Rt., retention time.

#### 3.3.1. X-376 fragmentation pattern

**Fig. 4:**
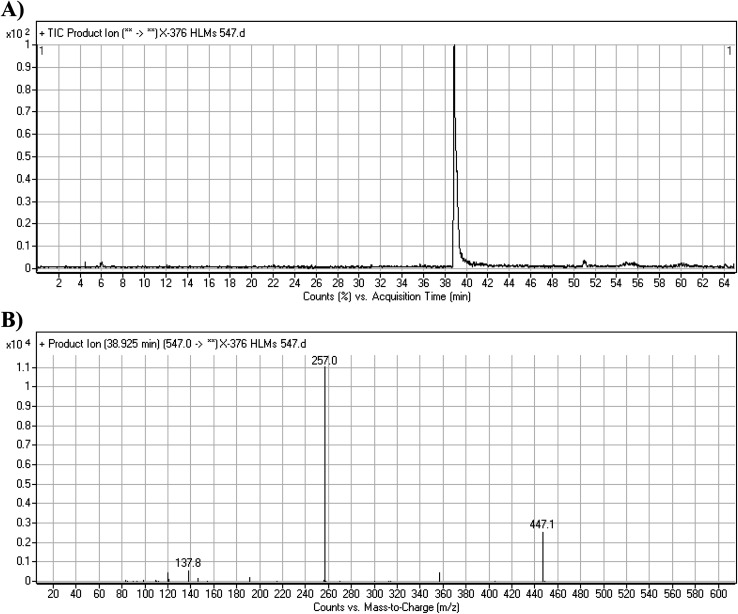
Product ion chromatogram at *m*/*z* 547 showing the X-376 peak at 38.9 min (A). Product ion mass spectrum of X-376 (B).

**Scheme 1:**
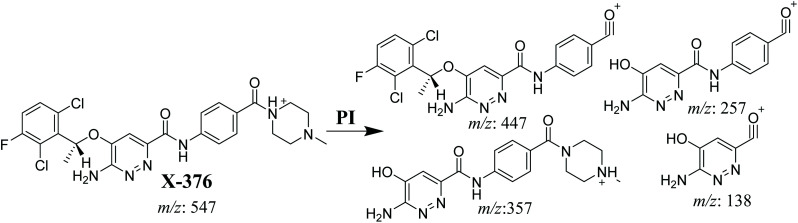
X-376 MS/MS fragments.

The X-376 molecular ion peak (MIP) was eluted at 38.9 min in the total ion chromatogram (TIC) (Fig. 4A). Collision induced dissociation (CID) of the molecular ion at *m*/*z* 547 generated four characteristic DIs at *m*/*z* 447, 357, 257, and 138, indicating reactive bonds inside the X-376 chemical structure (Fig. 4B and Scheme 1). The DI at *m*/*z* 447 represents the loss of the *N*-methyl piperazine ring. The DI at *m*/*z* 357 represents the loss of the 2,6-dichloro-3-fluorophenyl ring. Two other DIs at *m*/*z* 257 and *m*/*z* 138 have qualitative importance in the structural reconstruction of the metabolites.

#### 3.3.2. M1 fragmentation pattern

**Fig. 5:**
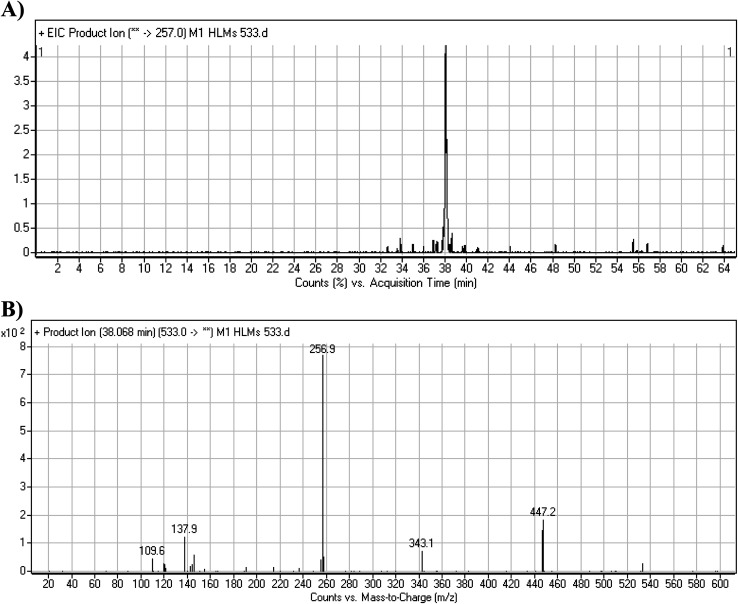
Product ion chromatogram at *m*/*z* 533 showing the M1 peak at 38.1 min (A). Product ion mass spectrum of M1 (B).

**Scheme 2:**
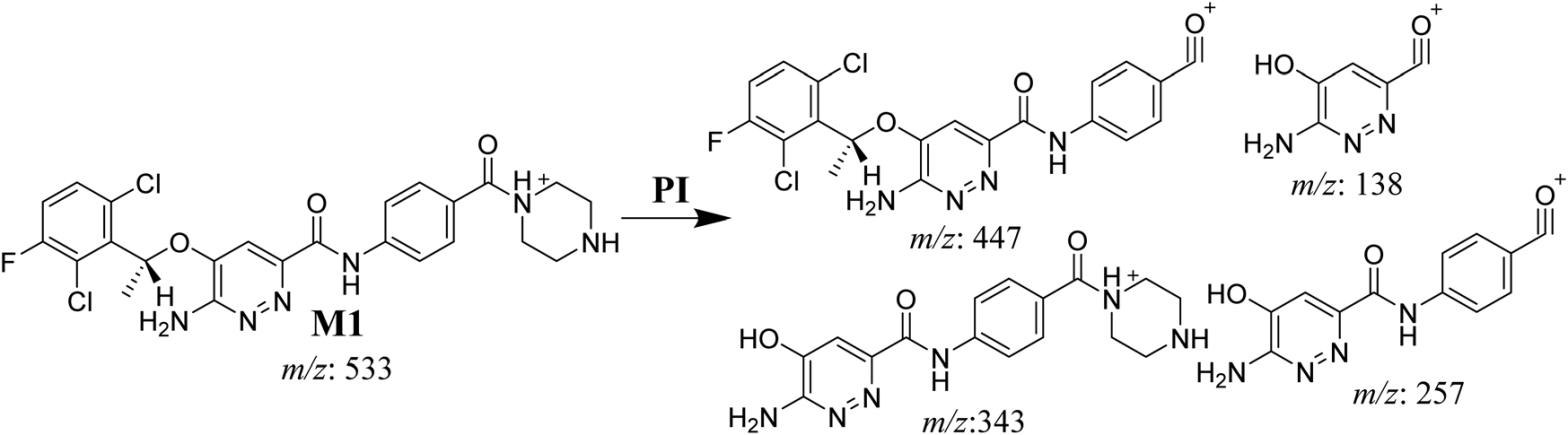
M1 MS/MS fragments.

The M1 MIP was eluted at 38.1 min in the TIC (Fig. 5A). The CID of the molecular ion at *m*/*z* 533 formed four DIs at *m*/*z* 447, 343, 257, and 138 (Fig. 5B). When compared to the X-376 fragments, the DI at *m*/*z* 343 revealed the *N*-demethylation metabolic reaction at the *N*-methyl piperazine ring due to the matching DIs at *m*/*z* 447, 257, and 138 (Scheme 2).

#### 3.3.3. M2 fragmentation pattern

**Fig. 6:**
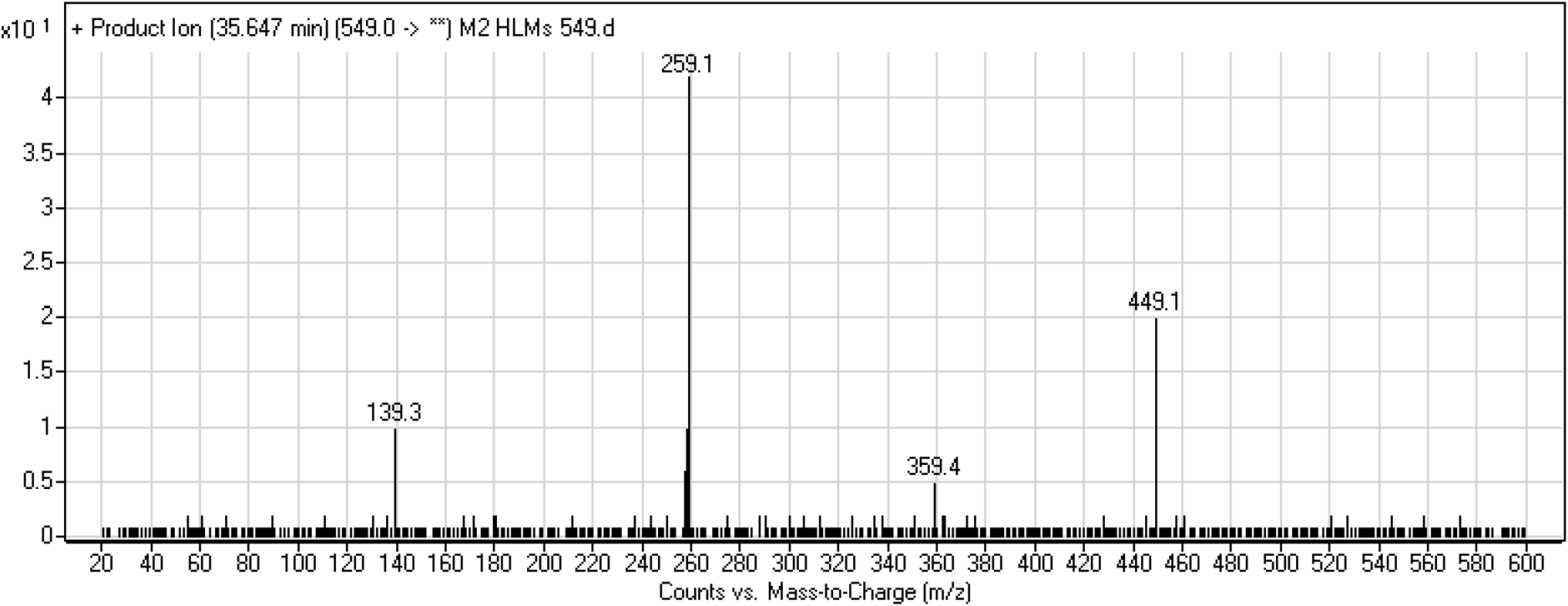
Product ion mass spectrum of M2.

**Scheme 3:**
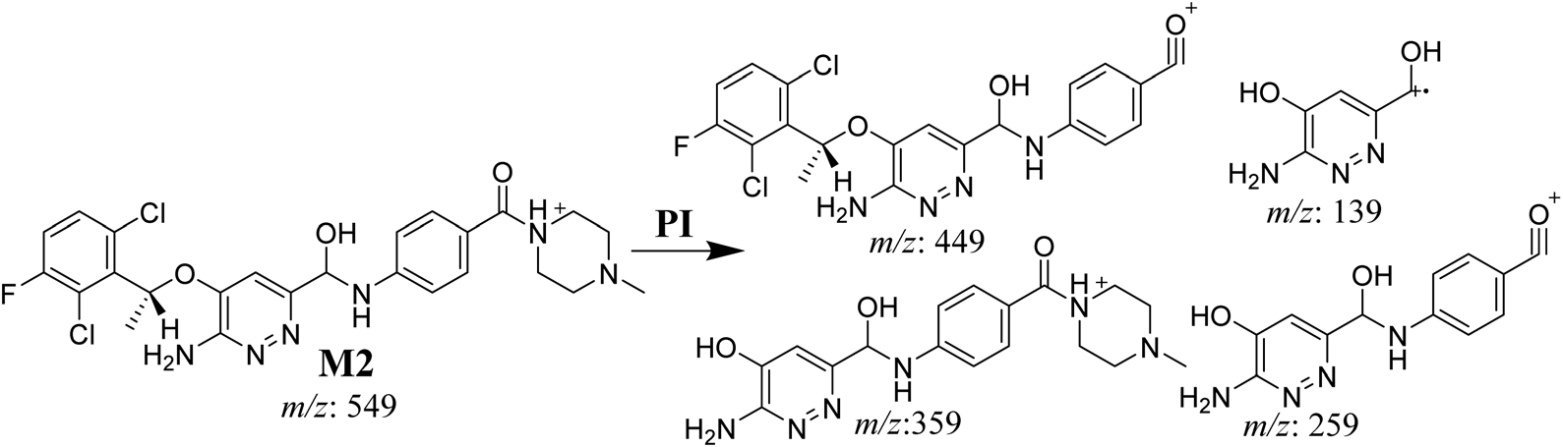
M2 MS/MS fragments.

The M2 MIP was eluted at 35.6 min in the TIC. The CID of the molecular ion at *m*/*z* 549 formed four DIs at *m*/*z* 449, 359, 259, and 139 (Fig. 6). When compared to X-376, the fragments of M2 generated DIs with an increase of 2 *m*/*z*. The proposed metabolic reaction was the reduction of amide group attached to the pyridazine ring that matched with the DI at *m*/*z* 139 (Scheme 3).

#### 3.3.4. M3 fragmentation pattern

**Fig. 7:**
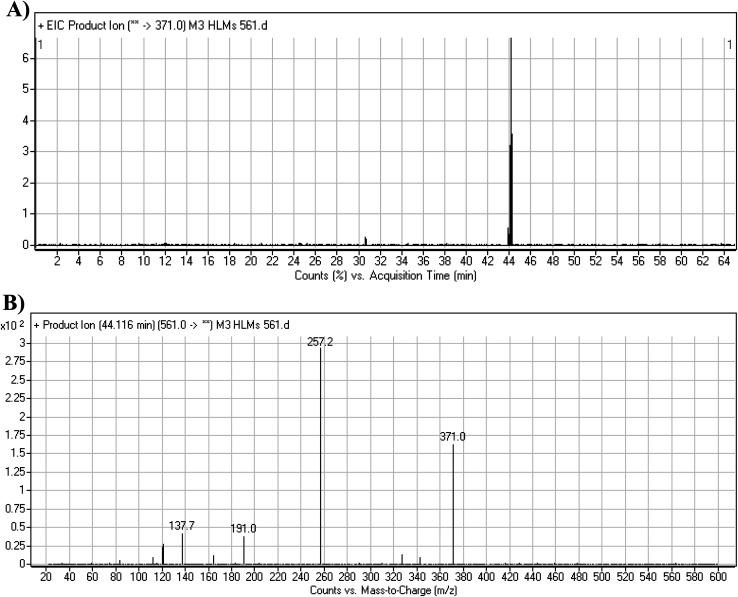
Product ion chromatogram at *m*/*z* 561 showing the M3 peak at 44.1 min (A). Product ion mass spectrum of M3 (B).

**Scheme 4:**
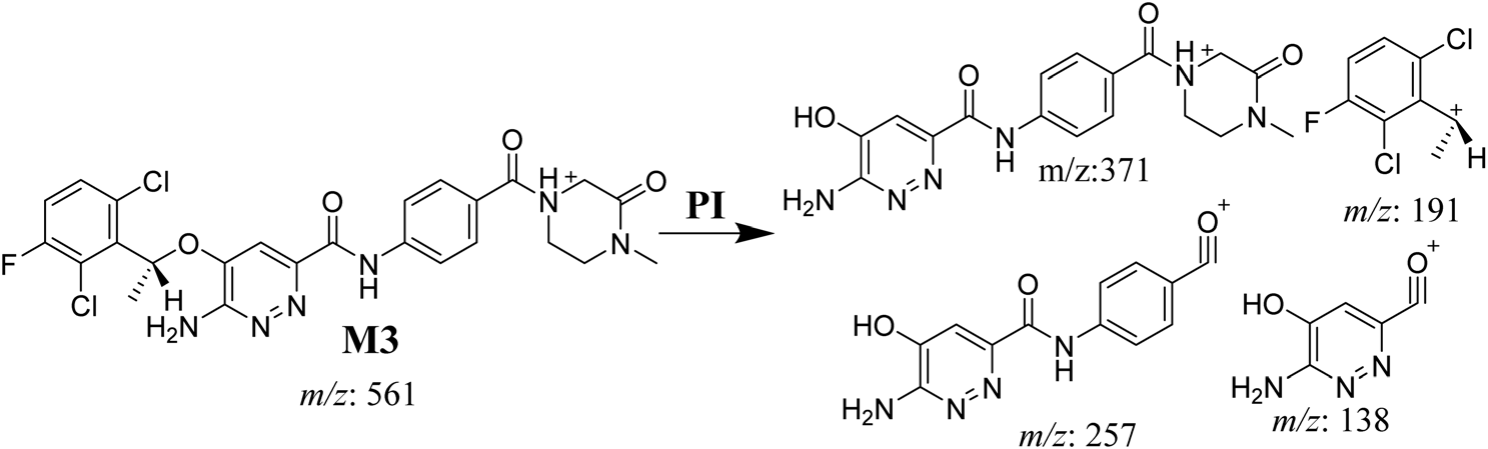
M3 MS/MS fragments.

The M3 MIP was eluted at 44.1 min in the TIC (Fig. 7A). The CID of the molecular ion at *m*/*z* 561 formed three DIs at *m*/*z* 371, 257, and 138 (Fig. 7B). If compared to the X-376 fragments, the DI at *m*/*z* 371 was indicative of oxidation of the *N*-methyl piperazine group, which was confirmed by the other two DIs at *m*/*z* 257 and 138 (Scheme 4).

#### 3.3.5. M4 fragmentation pattern

**Fig. 8:**
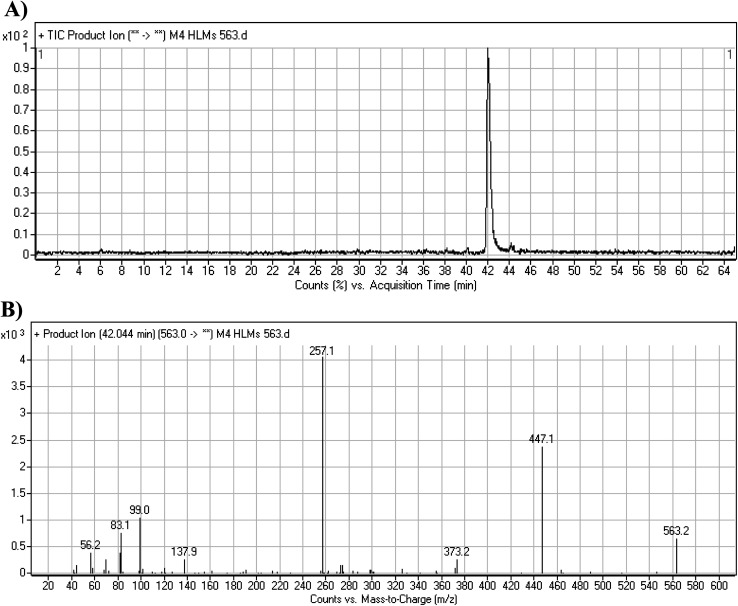
Product ion chromatogram at *m*/*z* 563 showing the M4 peak at 42.0 min (A). Product ion mass spectrum of M4 (B).

**Scheme 5:**
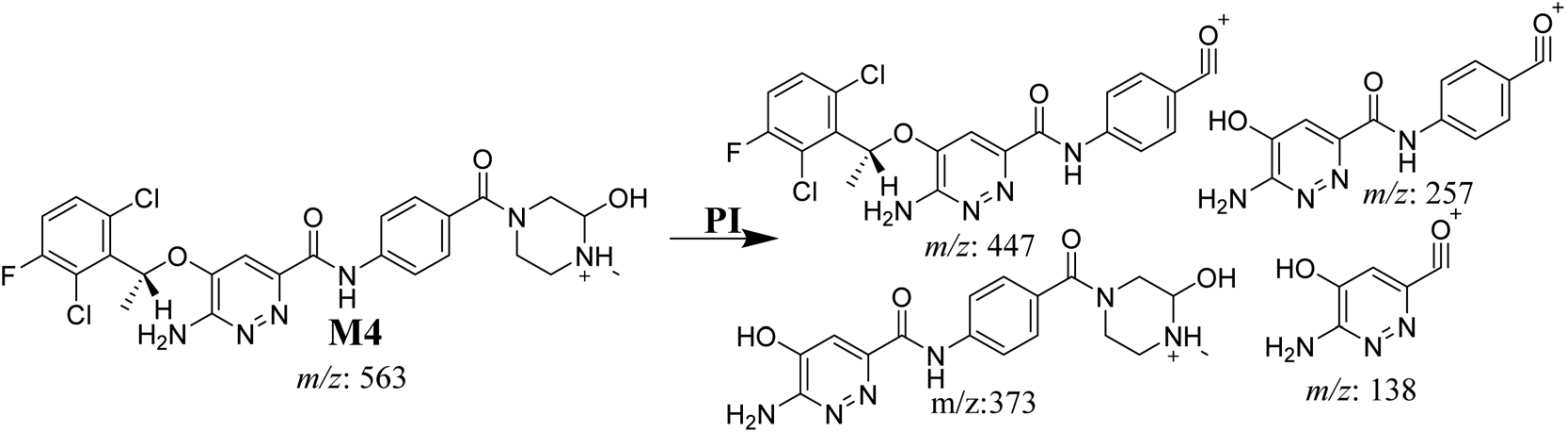
M4 MS/MS fragments.

The M4 MIP was eluted at 42.0 min in the TIC (Fig. 8A). The CID of the molecular ion at *m*/*z* 563 formed four DIs at *m*/*z* 447, 373, 257, and 138 (Fig. 8B). When compared to the X-376 fragments, the DI at *m*/*z* 373 was indicative of a hydroxylation metabolic pathway at the *N*-methyl piperazine group, which was verified by the two DIs at *m*/*z* 447 and 257 (Scheme 5).

### 3.4. Reactive metabolites

Two cyano and one GSH adduct were identified after incubation of X-376 with HLMs and capturing agents (KCN or GSH).

#### 3.4.1. M5 cyano adduct fragmentation pattern

**Fig. 9:**
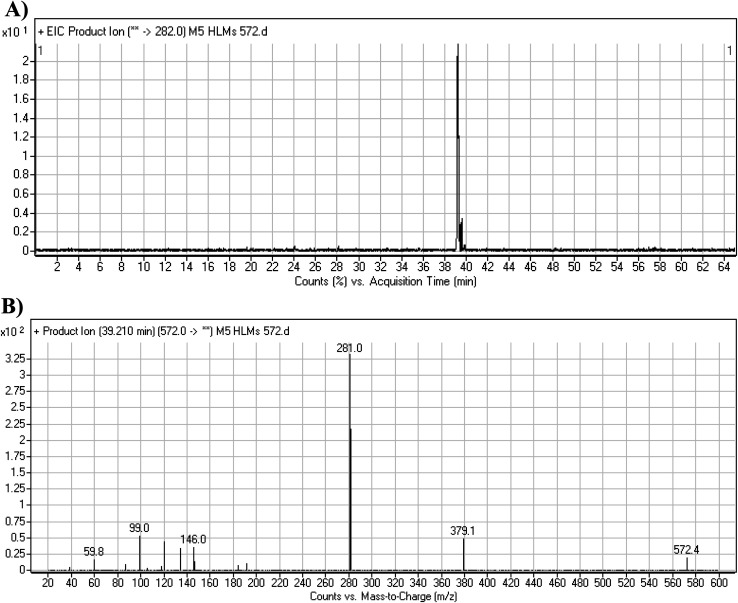
Product ion chromatogram at *m*/*z* 572 showing the M5 peak at 39.2 min (A). Product ion mass spectrum of M5 (B).

**Scheme 6:**
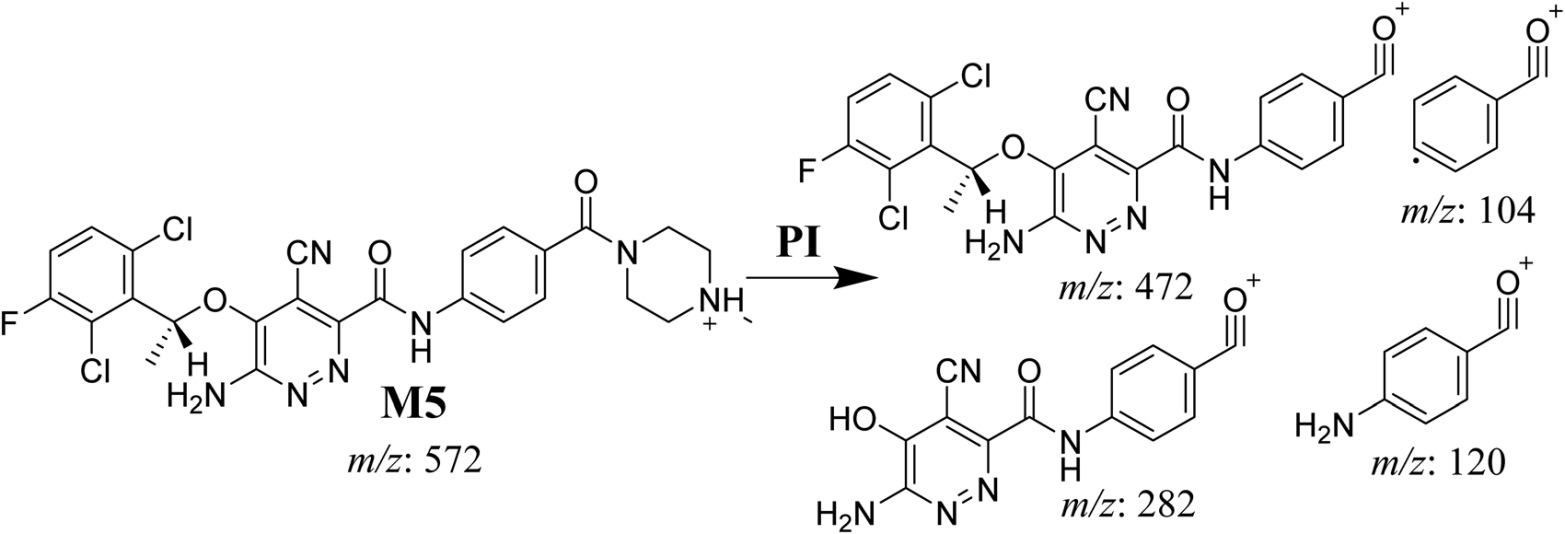
M5 MS/MS fragments.

The M5 MIP was eluted at 39.2 min in the TIC (Fig. 9A). The CID of the molecular ion at *m*/*z* 572 formed four DIs at *m*/*z* 472, 282, 120, and 104 (Fig. 9B). When compared to the X-376 fragments, the DIs at *m*/*z* 472 and 282 exhibited an increase of 25 *m*/*z* units, which revealed the addition of a cyano group at the bioactivated carbon of the pyridazine ring (Scheme 6).

#### 3.4.2. M6 cyano adduct fragmentation pattern

**Fig. 10:**
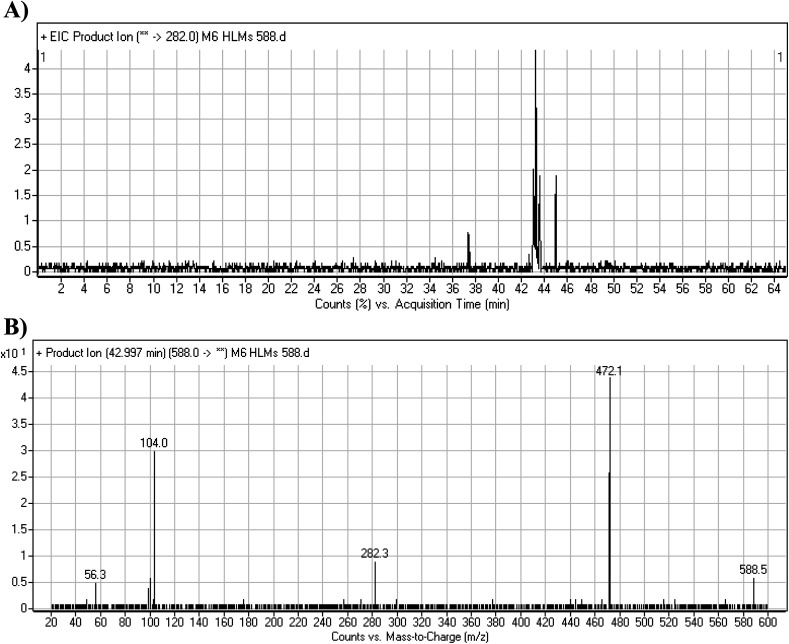
Product ion chromatogram at *m*/*z* 588 showing the M6 peak at 42.9 min (A). Product ion mass spectrum of M6 (B).

**Scheme 7:**
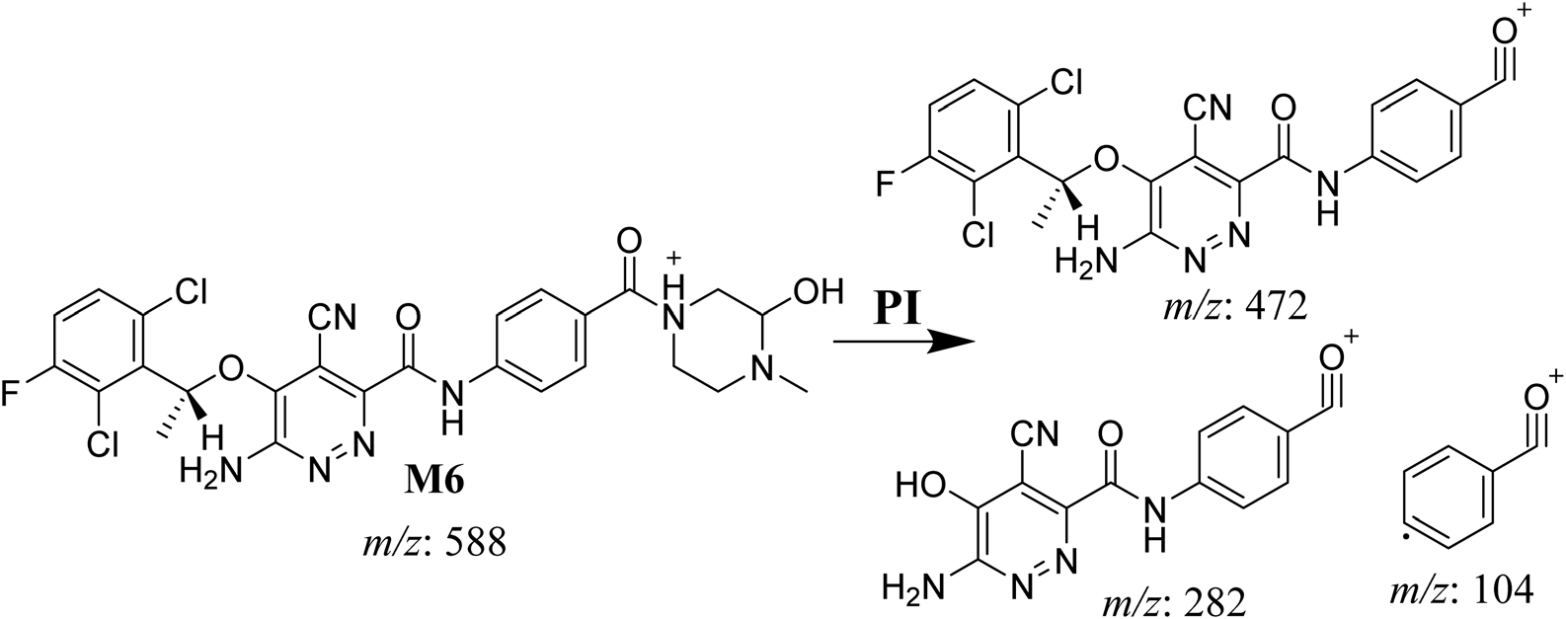
M6 MS/MS fragments.

The M6 MIP was eluted at 42.9 min in the TIC (Fig. 10A). The CID of the molecular ion at *m*/*z* 588 formed four DIs at *m*/*z* 472, 282, 120, and 104 (Fig. 10B). When compared to the X-376 fragments, the DIs at *m*/*z* 472 and 282 exhibited an increase of 25 *m*/*z* units, which revealed the addition of a cyano group. The metabolic pathways for M6 were hydroxylation at the piperazine ring and cyano addition at the bioactivated carbon of the pyridazine ring (Scheme 7).

#### 3.4.3. M7 GSH conjugate fragmentation pattern

**Fig. 11:**
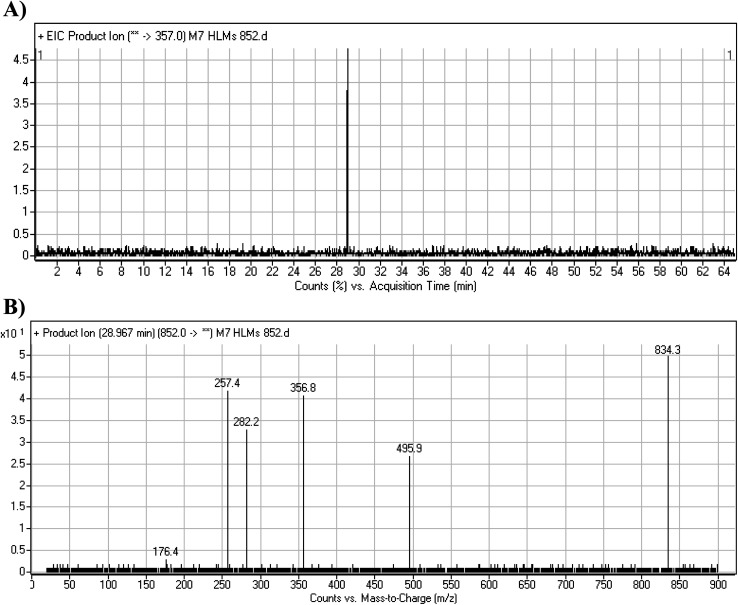
Product ion chromatogram at *m*/*z* 852 showing the M7 peak at 28.9 min (A). Product ion mass spectrum of M7 (B).

**Scheme 8:**
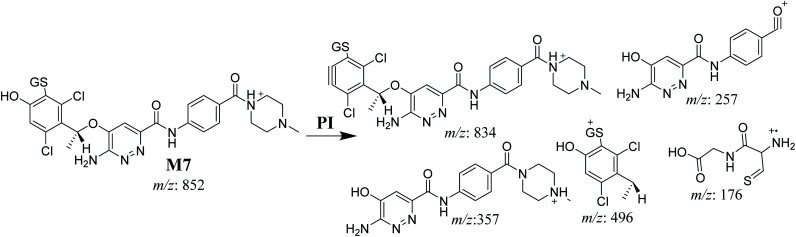
M7 MS/MS fragments.

The M7 MIP was eluted at 28.9 min in the TIC (Fig. 11A). The CID of the molecular ion at *m*/*z* 852 formed four DIs at *m*/*z* 496, 257, 357, and 176 (Fig. 11B). When compared to the ESB fragments, the DIs at *m*/*z* 357 and 257 confirmed that the bioactivation and conjugation with GSH occurred at the dichlorophenyl group after defluorination and hydroxylation (Scheme 8). This mechanism of bioactivation has been proposed for similar drugs (*e.g.*, diclofenac).^27^

### 3.5. Mechanism of X-376 bioactivation

**Scheme 9:**
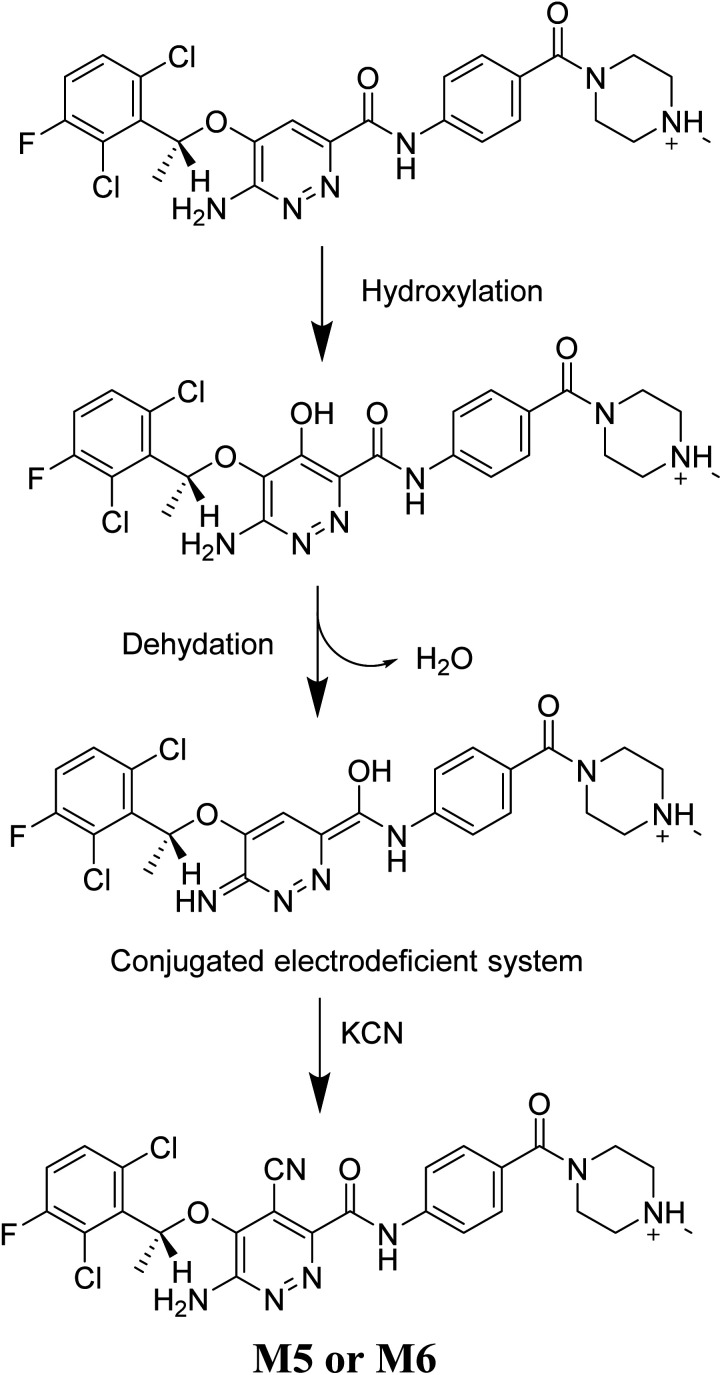
Supposed pathways for reactive iminium intermediate generation in X-376 metabolism and the possible capturing strategy.

The generation of M5 and M6 cyanide adducts revealed the production of reactive iminium intermediates in the *in vitro* metabolism of X-376. The hydroxylation metabolic pathway at the bioactivated pyridazine ring in X-376, followed by dehydration, created unstable iminium intermediates but they could be trapped as stable adducts with the cyanide nucleophile (Scheme 9). The supposed mechanism of the iminium intermediate generation and X-376 bioactivation pathway has been previously reported on similar cyclic tertiary amine-containing drugs.^32–35^

**Scheme 10:**
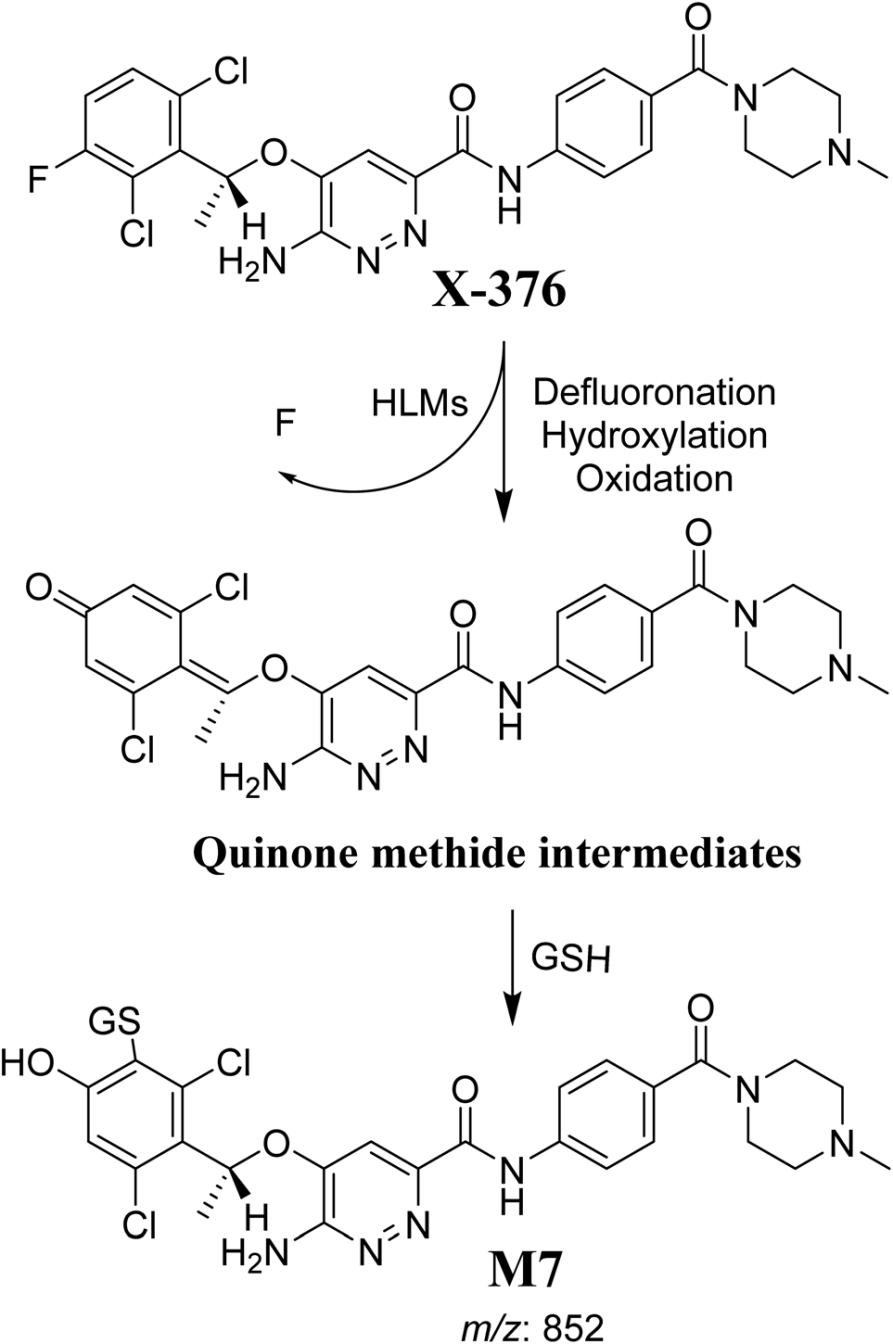
The proposed mechanism of GSH conjugation with quinone methide intermediate.

The formation of M7 revealed the production of a quinone methide reactive intermediate in X-376 metabolism, which was formed by defluorination, hydroxylation, and oxidation, and then trapped by GSH (Scheme 10).^27^

## 4. Conclusions

**Fig. 12:**
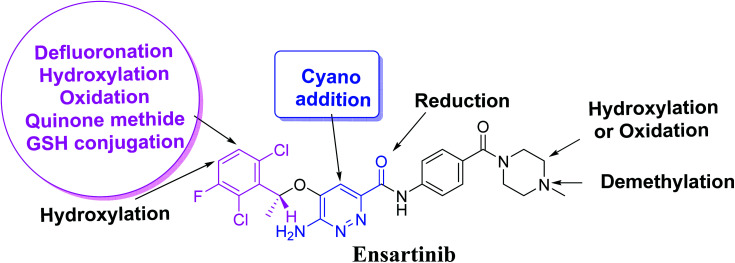
X-376 chemical structure indicating different metabolic phase I pathways and the corresponding bioactive centers.

**Fig. 13:**
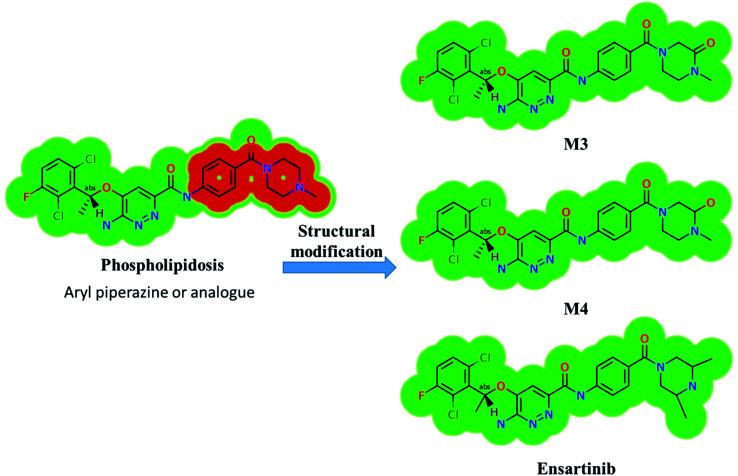
StarDrop DEREK module showing the disappearance of phospholipidosis structural alert in M3, M4, and ensartinib due to small structural modification in the aryl piperazine moiety.

Four metabolic reactions types, *viz.*, oxidation, *N*-demethylation, reduction, and hydroxylation, generated four *in vitro* phase-I X-376 metabolites. Bioactivation initiated the formation of two cyano adducts and one GSH conjugate (Fig. 12). The piperazine ring was not bioactivated as originally predicted. However, bioactivation occurred in the pyridazine ring because of the formation of an electron deficient system that could potentially bind to DNA or other important proteins inside the human body. The dichlorophenyl group was bioactivated by a novel pathway, which was confirmed using LC-MS/MS. These findings provide guidance for further work on X-376 toxicity. Further drug discovery studies can be done depending on this concept, thus allowing the development of new series of drugs with increased safety profile without affecting their pharmacological action. Blocking or adding isosteric substituents to the available bioactive pyridazine ring moiety would likely interrupt or block metabolic hydroxylation, which will stop the bioactivation sequence. The *in silico* toxicological studies for X-376 and its metabolites were performed using DEREK software, which revealed structural alerts and proposed side effects of X-376. Phospholipidosis was stopped as proposed by StarDrop DEREK module using small structural modifications, as in the case of M3, M4, and ensartinib (Fig. 13).

## Ethics

The study's design (*in vitro* assays using commercially available liver microsomes) exempts it from the approval by Ethics Committees.

## Authors' contributions

AAK and ASA supervised and designed the study. ASA, MWA, and AAK performed the optimization for the experimental steps and protocol. MWA conducted the experiments and drafted the manuscript. All the authors approved the final written draft of the manuscript.

## Conflicts of interest

The authors declare no competing interests.

## Supplementary Material
